# EBV-Driven Lymphoproliferative Disorders and Lymphomas of the Gastrointestinal Tract: A Spectrum of Entities with a Common Denominator (Part 1)

**DOI:** 10.3390/cancers13184578

**Published:** 2021-09-12

**Authors:** Magda Zanelli, Francesca Sanguedolce, Andrea Palicelli, Maurizio Zizzo, Giovanni Martino, Cecilia Caprera, Valentina Fragliasso, Alessandra Soriano, Luca Valle, Stefano Ricci, Alberto Cavazza, Francesco Merli, Stefano A. Pileri, Stefano Ascani

**Affiliations:** 1 Pathology Unit, Azienda USL-IRCCS di Reggio Emilia, 42123 Reggio Emilia, Italy; andrea.palicelli@ausl.re.it (A.P.); stefano.ricci@ausl.re.it (S.R.); alberto.cavazza@ausl.re.it (A.C.); 2 Pathology Unit, Policlinico Riuniti, University of Foggia, 71122 Foggia, Italy; francesca.sanguedolce@unifg.it; 3 Surgical Oncology Unit, Azienda USL-IRCCS di Reggio Emilia, 42123 Reggio Emilia, Italy; maurizio.zizzo@ausl.re.it; 4 Pathology Unit, Azienda Ospedaliera Santa Maria di Terni, University of Perugia, 05100 Terni, Italy; gio.martino@gmail.com (G.M.); ceciliacaprera@libero.it (C.C.); s.ascani@aospterni.it (S.A.); 5 Laboratory of Translational Research, Azienda USL-IRCCS di Reggio Emilia, 42123 Reggio Emilia, Italy; valentina.fragliasso@ausl.re.it; 6 Department of Pathology, Case Western Reserve University, Cleveland, OH 44106, USA; alessandra.soriano@ausl.re.it; 7 Gastroenterology Division, Azienda USL-IRCCS di Reggio Emilia, 42123 Reggio Emilia, Italy; 8 Anatomic Pathology, Department of Integrated Surgical and Diagnostic Sciences (DISC), University of Genoa and Ospedale Policlinico San Martino, 16132 Genoa, Italy; valleluca9@gmail.com; 9 Hematology Unit, Azienda USL-IRCCS di Reggio Emilia, 42123 Reggio Emilia, Italy; francesco.merli@ausl.re.it; 10 Haematopathology Division, European Institute of Oncology-IEO IRCCS Milan, 20141 Milan, Italy; stefano.pileri@unibo.it

**Keywords:** Epstein–Barr virus, EBV-positive mucocutaneous ulcer, classic Hodgkin lymphoma, EBV-positive, diffuse large B-cell lymphoma, not otherwise specified

## Abstract

**Simple Summary:**

Epstein–Barr virus (EBV) infection usually occurs early in life. The virus persists throughout the lifespan in a latent phase mainly in B lymphocytes of immunocompetent hosts. Conditions of immunosuppression of variable origins may favor the emergence of EBV-linked lymphoid proliferations. This group of disorders, having EBV as common denominator, encompasses entities ranging from indolent diseases to aggressive lymphomas. In this review, consisting of three parts, we focus on EBV-linked lymphoid proliferations which may occur in the gastrointestinal tract. Our aim is to summarize the salient clinical, pathological, molecular and therapeutic data of this group of heterogeneous entities, often showing overlapping morphologic and immunophenotypic features despite the different clinical behavior. The correct diagnosis is essential in order to adopt the adequate treatment. In this part of the review, the available data on EBV biology, EBV-positive mucocutaneous ulcer, EBV-positive diffuse large B-cell lymphoma, not otherwise specified and classic Hodgkin lymphoma are discussed.

**Abstract:**

EBV is the most common persistent virus in humans. The interaction of EBV with B lymphocytes, which are considered the virus reservoir, is at the base of the life-long latent infection. Under circumstances of immunosuppression, the balance between virus and host immune system is altered and hence, EBV-associated lymphoid proliferations may originate. These disorders encompass several entities, ranging from self-limited diseases with indolent behavior to aggressive lymphomas. The virus may infect not only B-cells, but even T- and NK-cells. The occurrence of different types of lymphoid disorders depends on both the type of infected cells and the state of host immunity. EBV-driven lymphoproliferative lesions can rarely occur in the gastrointestinal tract and may be missed even by expert pathologists due to both the uncommon site of presentation and the frequent overlapping morphology and immunophenotypic features shared by different entities. The aim of this review is to provide a comprehensive overview of the current knowledge of EBV-associated lymphoproliferative disorders, arising within the gastrointestinal tract. The review is divided in three parts. In this part, the available data on EBV biology, EBV-positive mucocutaneous ulcer, EBV-positive diffuse large B-cell lymphoma, not otherwise specified and classic Hodgkin lymphoma are discussed.

## 1. Introduction

EBV is a ubiquitous virus, infecting the majority of the world population [1,2,3,4,5,6,7,8,9,10,11,12]. After primary infection, usually occurring at an early age, EBV remains persistently in the B-cells of most adults. Normally, the complex equilibrium between the virus and the host immune system is capable of maintaining EBV-infected B-lymphocytes at a low number, preventing the development of pathological lymphoid proliferations [2,3].

EBV-associated lymphoproliferative disorders (EBV-LPDs) may emerge when immunosuppression (IS) of different etiologies causes a disruption of the fine balance between the virus and host immune system. Primary immunodeficiency, HIV infection, the post-transplant setting and some types of immunosuppressive drugs are circumstances in which EBV-LPDs may occur [4].

A physiological process of decay of the immune system, named immunosenescence, variably affects individuals with aging. The process of immunosenescence is characterized by the reduced production of lymphoid precursors and altered T-cell immunity. With aging, there is a depletion of new, naïve CD8-positive T-cells, which are replaced by mature, memory T-cells with a defective function [5,6]. Similarly to other conditions of IS, immunosenescence favors EBV-LPDs development [5,6,7,8,9].

Since the discovery of the virus more than 50 years ago, EBV has been demonstrated to play a relevant role in the pathogenesis of different types of neoplasms, including LPDs. Besides B lymphocytes, EBV has the ability to infect T lymphocytes and natural killer (NK) cells and hence, it is involved in the emergence of EBV-associated LPDs of B-, T- and NK-cell origin [7,8,9].

EBV-driven LPDs represent a broad and expanding clinicopathological spectrum of diseases, ranging from indolent and often self-limiting disorders, such as EBV-positive mucocutaneous ulcer (EBVMCU), to aggressive lymphomas.

The aim of this review is to comprehensively overview EBV-LPDs arising in the gastrointestinal tract (GIT), focusing on the main differential diagnoses.

The review consists of three parts. In this first part, EBVMCU [8,13,14,15,16,17,18,19,20,21,22,23,24,25,26,27,28,29,30,31,32,33,34,35,36,37,38], EBV-positive diffuse large B-cell lymphoma not otherwise specified (EBV-positive DLBCL, NOS) [8,39,40,41,42,43,44,45,46,47,48,49,50,51,52,53,54,55] and classic Hodgkin lymphoma (cHL) [8] are discussed.

In part 2, plasmablastic lymphoma (PBL), extra-cavitary primary effusion lymphoma (EC-PEL) and Burkitt lymphoma (BL) are discussed; whereas in part 3, post-transplant lymphoproliferative disorders (PTLDs), chronic active EBV (CAEBV) infection of T- and NK-cell type and extranodal NK/T-cell lymphoma (ENKTL) nasal type are detailed [8].

## 2. Epstein–Barr Virus Biology

EBV is one of the most common human virus; it belongs to the Herpes virus family and is also known as Herpes virus 4 [1].

EBV is ubiquitous and is transmitted through saliva. Most individuals develop the infection during childhood or adolescence and the infection generally remains clinically silent; when primary infection is symptomatic usually follows a self-limited course manifesting as infectious mononucleosis (IM) [10].

During EBV infection, there are two phases: a lytic phase and a latent phase [7,11,12]. EBV is capable of infecting both epithelial cells and B lymphocytes. During the lytic phase, the virus replicates in the oropharynx epithelium. During the latent phase, the viral genome persists in the oropharyngeal lymphoid tissue.

The mechanism of EBV entry into B lymphocytes is based on the interaction of the major viral surface glycoprotein (gp350) with its primary receptor on B lymphocytes, so-called complement receptor 2 (CR2/CD21) or CR1 (CD35). A second viral glycoprotein (gp42) binds the class II major histocompatibility complex molecules on B lymphocytes functioning as coreceptors.

In healthy hosts, lymphoid B-cells, particularly memory B-cells, represent the EBV reservoir. To escape T-cell immune-surveillance, latent viral proteins are not expressed in memory B-cells and EBV genome remains life-long within B-cells in the episomal form.

In the latent phase, EBV gene products upregulate the expression of various B-cell genes. EBV-infected cells express six types of EBV nuclear antigen (EBNA)—EBNA1, EBNA2, EBNA3A, EBNA3B, EBNA3C and EBNA leader protein—and three types of latent membrane protein (LMP), LMP1, LMP2A and LMP2B.

Depending on the specific viral expression pattern, there are three types of latency phase of EBV infection [2]. In Latency I, only EBNA1 is expressed in all infected cells; EBNA1 is the cause of viral genome maintenance and replication. Latency II is a phase of transition characterized by the expression of various proteins such as LMP1, LMP2A and LMP2B. Latency II consists of Latency IIa and Latency IIb. Latency IIa is characterized by the expression of LMP1, LMP2A and EBNA1 and it is a phase of transition to Latency III. In Latency IIa, the infected cells acquire the ability to avoid cytotoxic T lymphocytes. In Latency IIb, there is EBNA2 expression, in absence of LMP1; in Latency IIb, infected cells prepare for transition to Latency III, in which all gene products are expressed.

The BamHI Z EBV replication activator (ZEBRA) mediates disruption of latency and induction of EBV early gene expression in latently infected lymphocytes.

EBV is etiologically related to several neoplasms, including gastric carcinoma, nasopharyngeal carcinoma and different types of lymphoma [1,2].

EBV employs different mechanisms to evade the host immune response system in order to survive. The programmed cell death protein 1 (PD-1) is an immune checkpoint regulating the host immune response. The binding of PD-1 ligand 1 (PD-L1) on neoplastic cells to PD-1 on T-lymphocytes produces inhibitory signals to T-cells, allowing the tumor cells to evade the host immune system [50,51,52,53,54,55]

Recent data have demonstrated that PD-L1 expression is a common feature of EBV-linked LPDs, supporting the concept that these diseases may benefit from checkpoint inhibitor treatment, blocking the interplay between PD-1 of T lymphocytes and PD-L1 on neoplastic cells and, hence, enhancing anti-tumor immune response [56,57].

It remains not completely elucidated which viral-encoded proteins may affect PD-L1 expression and how they act, although recent studies have shown that EBV alters PD-L1 expression mainly through EBNA2 [58]. Therefore, EBNA2-positive lymphomas may have a better prognosis with immune checkpoint blockers. Further studies are required to fully explore this interesting and therapeutically relevant issue.

## 3. EBVMCU

### 3.1. General Features and Etiology

EBVMCU was first described in 2010 by Dojcinov et al. [13] in a series of EBV-positive, circumscribed and superficial mucosal and cutaneous ulcers arising in different setting of IS, including old age.

EBVMCU involves extra-nodal sites, occurring in the oropharynx (52%), skin (29%) and GIT (19%) [13,14,15,16,17,18,19,20,21,22,23,24,25,26,27,28,29,30].

In the current 2017 WHO classification, EBVMCU is identified as a provisional clinicopathological entity strictly linked to EBV [8].

Although its histology can be reminiscent of more aggressive lymphomas, such as cHL and EBV-positive DLBCL-NOS, the disease course is mostly indolent, with regression upon removal of the immunosuppressive cause. The cell of origin of EBVMCU is considered to be an EBV-transformed lymphoid B-cell [13].

EBVMCU develops in the context of iatrogenic IS (56–66%), in advanced age favored by immunosenescence (27% to 40% of cases) or in primary immunodeficiency (2% to 4% of cases) [14]. It has been reported in patients on immunosuppressive treatments such as methotrexate (MTX), cyclosporin A (CYA), Tacrolimus (Tac), prednisolone (PSL), azathioprine (AZA) or TNF-alpha inhibitors for autoimmune conditions or for solid organ/bone marrow transplantation [1,13,14,15,16,17,18,19,20,21,22,23,24,25,26,27,28,29,31,32,33,34,35,36,37,38]. EBVMCU is also reported after another lymphoma or tumor treatment [30].

The disease may emerge when the virus overwhelms the host immune response. Its hallmark is the localized and superficial nature of the ulcer, in absence of a tumor-forming lesion.

Noteworthy, the presence of a mass and/or involvement of lymph nodes, liver, spleen and bone marrow (BM) substantially excludes the diagnosis of EBVMCU [13].

The frequent localization in the oropharynx (69.3% of cases) and, less commonly, in the skin and GIT, is probably due to the release of EBV into saliva.

In immunocompromised hosts, conditions favoring EBVMCU may be represented either by a local trauma (for instance, tooth extraction) or chronic mucosal irritation [13,15]. The superficial nature of the disease and its indolent course might be explained by the occurrence of a slight and localized lapse in immune-surveillance over EBV.

### 3.2. EBVMCU and GIT

The occurrence of EBVMCU in GIT is rather uncommon, with just about 30 cases reported so far [13,17,18,19,20,21,22,23,24,27,28,29,30,31,32,33,34,35,36,37,38].

The most common setting is iatrogenic IS in inflammatory bowel disease (IBD) patients [13,18,19,20,28], followed by solid organ-transplanted patients [13,16,17], immune-related colitis (irColitis) [29], rheumatoid arthritis [13] and treated lymphoma [30]. Occasional cases are reported in patients with autoimmune thrombocytopenia, HIV, post-hematopoietic stem cell transplantation, hypogammaglobulinemia and rheumatic polymyalgia [21,22,37]. In a minority of GIT cases, EBVMCU develops in advanced age, without other known causes of IS [16,23,24].

In the majority of cases, iatrogenic IS either by AZA, infliximab (IFX), 6-mercaptopurine, CYA, MTX, mycophenolate mofetil (MMF), PSL and Tac is present [37].

The colon is the most commonly involved site [37].

The local irritative stimulus favoring EBVMCU occurrence may be represented by chronic mucosal inflammation in IBD [13,18,19,20,28] and irColitis [29] and even by large bowel inflamed diverticula [21,24].

The lesions are typically single, although multiple lesions are reported in IBD [19,28], in irColitis [29] and in colon diverticula [24].

EBVMCU superimposed on IBD clinically presents as a refractory IBD [20]. Its correct identification is of paramount importance, as refractory IBD is usually treated with a strong immunosuppressive therapy, whereas EBVMCU may regress with removal of iatrogenic immunosuppression [25].

Interestingly, GI EBVMCU have been recently reported in the setting of irColitis, a type of colitis related to the use of immunotherapies targeting the immune checkpoints T-lymphocyte-associated protein 4 (CTLA-4) and PD-1.

irColitis-related EBVMCU often consists of multiple ulcerating lesions, which may be complicated by colon perforation, an uncommon event in patients with EBVMCU in other settings [29,37]. In the correct clinical context, ulcerating lesions leading to perforation may be considered the hallmark of irColitis-related EBVMCU [29,37].

### 3.3. Histology, Immunophenotype and Genetic Profile

The ulcers are superficial and circumscribed [13]; in rare cases, multiple ulcers are reported [24,29].

On histology, the ulcers consist of EBV-positive atypical cells in the context of a polymorphic infiltrate including inflammatory cells (granulocytes, histiocytes, plasma cells) and are often associated with necrosis and apoptotic bodies.

In situ hybridization for EBV-encoded RNA (EBER) is diffusely positive. Typically, the EBV-positive cells are of different sizes, from small to large cells with Hodgkin and Reed Sternberg-like features. Angioinvasion by EBV-positive cells is a frequent finding.

The histological spectrum of EBVMCU is variable; some cases, with a more polymorphic pattern, simulate cHL and others, with a monomorphic and diffuse pattern of growth, mimic EBV-positive DLBCL-NOS [15,26].

A rather characteristic rim of small CD8 positive T lymphocytes is usually present at the base of the ulcer.

The atypical EBV-positive cells usually show strong CD30 positivity with variable expression of B-cell markers such as CD20 and CD79 alpha. PAX5, MUM1/IRF4 and the transcription factor OCT2 are usually positive, whereas BOB1 is variably expressed. CD15 is positive in 50% of cases. CD10 and BCL6 are generally negative. LMP1 is usually positive with Latency pattern IIa or III.

Clonality may be detected. About 40% of cases may harbor clonal immunoglobulin heavy chain (IGH) gene rearrangement and/or T-cell receptor (TCR) gene rearrangement [13]. Cases with T-cell clonality are more often described in the setting of age-related IS [13,14]. The presence of clonality is supposed to be the consequence of the clonal selection caused by EBV.

### 3.4. Differential Diagnosis

EBVMCU may display features overlapping with cHL and EBV-positive DLBCL, NOS.

Primary GI cHL is a rare event and a diagnosis of primary cHL at extra-nodal sites, including GIT, should always be made with caution.

Unlike EBVMCU, which is superficial, primary GI cHL is generally a tumor-forming lesion with transmural GI involvement [13,21]. Morphology and immunohistochemical profile of cHL are similar to EBVMCU.

Helpful histological clues observed in EBVMCU and not in cHL are the high number of EBV-positive cells of variable sizes and the small rim of CD8-positive T lymphocytes at the base of the ulcer [13].

EBV-positive DLBCL, NOS may involve both lymph nodes and extra-nodal sites, including the GIT. It usually forms a mass, diffusely effacing the tissue architecture, unlike EBVMCU. Histologically, EBV-positive DLBCL, NOS can show a monomorphic appearance quite easy to identify due to similarity with conventional diffuse large B-cell lymphoma (DLBCL). Cases of EBV-positive DLBCL, NOS with a polymorphic pattern of growth, containing Hodgkin-like cells, display features reminiscent of both cHL and EBVMCU.

In EBV-positive DLBCL, NOS, CD30 is often positive, with less frequent co-expression of CD15. Unlike EBVMCU and cHL, EBV-positive DLBCL, NOS shows a full B-cell phenotype, with expression of CD20, CD19, PAX5, CD79alfa and the transcription factors BOB1 and OCT2 are usually positive [8,9,13]. Similarly to EBVMCU, EBV-positive DLBCL, NOS has as an activated phenotype, with positivity for MUM1/IRF4 and negativity for CD10 [8]. In order to differentiate these EBV-positive LPDs, it is of paramount importance to know the whole clinicopathological picture and particularly, the extent of the disease.

For completeness, it has to be mentioned that angioinvasion and necrosis often observed in EBVMCU may histologically resemble the angiocentric features characteristic of lymphomatoid granulomatosis (LYG) [8]. Unlike EBVMCU, LYG usually presents with nodules and lung is the most commonly involved site.

### 3.5. Treatment and Outcome

EBVMCU is characterized by a self-limited course, with either spontaneous remission or regression upon reduction or removal of the immunosuppressive therapy [13,15].

Only in a few cases do the patients experience a recurrence or a progressive disease. Rare cases require a therapeutic intervention, including standard radiotherapy and chemotherapy [13,26,27,38]; rituximab as single agent is reported to be effective [13,17,28].

Patients with treated-lymphoma-associated EBVMCU have been observed to have a worse behavior than patients with MTX-associated EBVMCU due to lymphoma relapse or unrelated causes [30]; therefore, EBVMCU emergence in patients treated for lymphoma may predict an adverse clinical course [30,37].

Similarly to EBVMCU arising in other sites, GI EBVMCU often follows an indolent behavior, with remission upon reduction or suspension of IS.

Progression to overt lymphoma represents a rare event as in the report of Moran et al. presenting a case of EBVMCU arising in the setting of iatrogenic IS for Crohn’s disease, in which the patient experienced progression to widespread cHL [19].

In IBD patients, the treatment strategy for IBD itself after regression of EBVMCU is not well defined, as reintroduction of immunosuppressive treatment may cause relapse of the EBV-positive LPD [37]. irColitis-related EBVMCU often leads to perforation requiring surgical resection; however, the clinical course after surgery is usually favorable [29,37].

## 4. EBV-Positive DLBCL, NOS

### 4.1. General Features and Etiology

EBV-positive DLBCL, NOS is a clonal B-cell neoplasm initially described in patients older than 50 years, often presenting with an extra-nodal and highly aggressive disease [39,40].

In the 2008 WHO classification, the disease was named EBV-positive DLBCL of the elderly [41]. The required diagnostic features included age over 50 years, absence of any known condition of IS and no prior history of lymphoma.

However, the identification of the disease even in younger adults (<50 years) and in children led to change the terminology in the current 2017 WHO classification [8,39,40,41,42,43,44,45,46,47,48].

The disease is worldwide, although prevalent in Asia and South America (5–15% of all DLBCLs) and rare in Europe (<5%) [39]. Most cases involve individuals aged over 50 years, with a median age of 71 years and a slight male predominance [8].

The disease can involve both nodal and extra-nodal sites.

The nodal presentation is more common in younger patients, generally having lower stage and better prognosis than older patients [42,43].

The form affecting elderly individuals is observed predominantly at extra-nodal sites, including skin, lung and GIT, and patients often exhibit B symptoms and have a poor outcome [8,39,40,44].

The disease is EBV-driven both in younger and older patients and is generally characterized by a type II latency pattern with downregulation of EBNA2 and upregulation of LMP1. In a minority of cases, there is a type III latency pattern with EBNA2 upregulation. Type III latency pattern is associated with a marked immunodeficiency state and is commonly seen in PTLDs and in HIV-associated lymphomas [8,25,42].

### 4.2. EBV-Positive DLBCL, NOS and GIT

In general, the main criterion to differentiate primary GI lymphomas from a systemic disease involving the GI tract is represented by the localization of the main bulk of the tumor in GIT.

Clinical and prognostic differences have been observed between EBV-positive DLBCL, NOS localized in the stomach and the intestinal form [44].

The gastric variant seems to occur more often in immunocompetent individuals and the disease is usually localized with better outcome [44].

The intestinal form preferably arises in the context of IS and at a more advanced stage with a consequent worse prognosis [42,45]. The association of intestinal EBV-positive DLBCL, NOS with a state of immunological deterioration is rather striking, resembling EBV-positive DLBCL, NOS of the central nervous system (CNS), usually associated with conditions of IS [46].

### 4.3. Histology, Immunophenotype and Genetic Profile

The current 2017 WHO classification recognizes the monomorphic pattern and the polymorphic or T-cell/histiocyte-rich large B-cell lymphoma-like pattern, the latter being the most common in young patients [8].

The monomorphic pattern composed of sheets of large cells is difficult to distinguish from EBV-negative DLBCL, unless EBER is performed [8].

**Figure 1:**
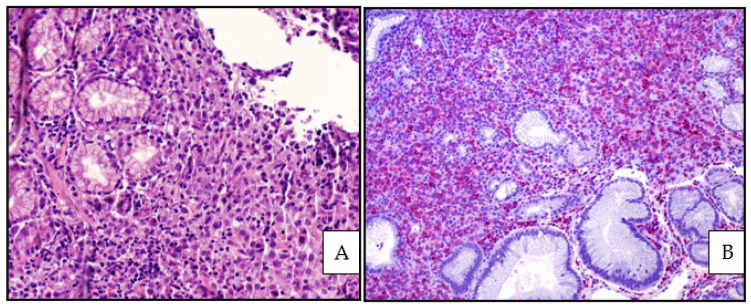
(**A**) EBV-positive DLBCL, NOS: medium power view showing a polymorphic infiltrate within the gastric mucosa (Hematoxylin and eosin, magnification 400×; original image from Prof S.A. (**B**) EBV-positive DLBCL, NOS: CD79alpha positivity highlighting the B-cell phenotype of the lymphoid infiltrate (immunostaining, magnification 200×; original image from Prof S.A.).

**Figure 2:**
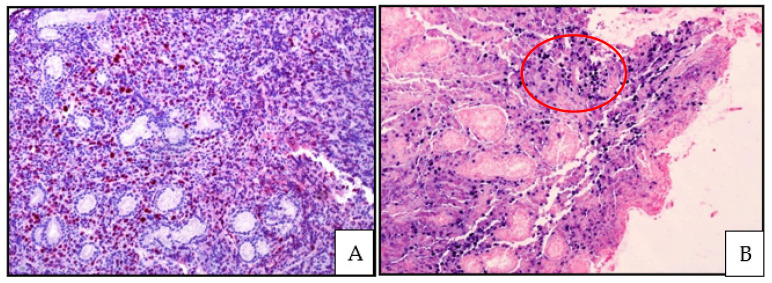
(**A**) EBV-positive DLBCL, NOS: Ki67 immunostaining revealing an elevated proliferative index of the lymphoid infiltrate (magnification 200×; original image from Prof S.A.); (**B**) EBV-positive DLBCL, NOS: EBER showing positive signals for EBV in the nuclei of the lymphoid infiltrate (inside the red circle) (magnification 200×; original image from Prof S.A.).

The polymorphic pattern consists of scattered large cells, some with Hodgkin- and Reed Sternberg-like features, within a reactive microenvironment including histiocytes, small lymphocytes and plasma cells (Figure 1 and Figure 2).

Areas of geographical necrosis and angioinvasion may be observed [8,47]. Neoplastic cells express pan B-cell markers (CD20, CD79 alpha, PAX5, CD22, CD19) and have a non-GCB phenotype, being positive for MUM1/IRF4 and negative for CD10 and commonly BCL6. CD30 is often expressed, and CD15 co-expression more rarely. LMP1 is found in two-thirds of cases, while EBNA2 is found in one-third of cases, realizing a type II or type III latency patterns [8,47].

EBER expression in neoplastic cells is required for diagnosis. The extent of EBER positivity is debated, but a threshold of 80% for diagnosis is recommended in the current WHO classification [8,37,47]. Of note, the presence of few EBER-positive non neoplastic, bystander cells can be observed in other B-cell and T-cell lymphomas [8].

Clonal IGH rearrangement is usually detected and approximately 60% of cases show restricted or oligoclonal TCR gene rearrangements [40]. The genetic profile of EBV-positive DLBCL, NOS is different from the EBV-negative counterpart. EBV induces the activation of the Janus kinase signal-transducer and activator of transcription (JAK-STAT) and of nuclear factor kappa B (NF-κB) pathways [48]. Chromosomal gains at 9p24.1 contribute to increased expression of PD-L1 and PD-L2, favoring immune tolerance and tumor evasion and hence, poor outcome [8,47].

### 4.4. PD-L1 Expression

Immune evasion, mostly by PD-L1 and the PD-1 pathway, is an attractive therapeutic target in different types of neoplasms [49]. In conventional DLBCL, the frequency of the reported PD-L1 expression is variable (from 6% to 26%) depending on different cut-off values and different anti-PD-L1 monoclonal antibodies [50,51]. In DLBCL, PD-L1 is also expressed on cells of the microenvironment such as macrophages and dendritic cells [52]. It is still controversial whether PD-L1 expression on tumor cells has an adverse prognostic significance [37,52,53]. In EBV-positive DLBCL, NOS, EBV increases PD-L1 promoter and enhancer activity [54]. Compared to the EBV-negative counterpart, in EBV-positive cases, there is a higher frequency of PD-L1 expression on both tumor cells and immune cells [52,53]. The expression of PD-L1 on tumor cells and microenvironment immune cells seems to have a divergent impact on prognosis. EBV-negative GI DLBCL with expression of PD-L1 on the microenvironment cells have a better outcome compared to PD-L1 negative cases [39]. EBV-positive GI DLBCL with PD-L1 expression on tumor cells have been observed to follow an aggressive clinical course [55].

### 4.5. Differential Diagnosis

In the GIT, the spectrum of EBV-associated LPDs to consider for differential diagnosis is rather wide, including PTLDs and EBVMCU.

The differential diagnosis with EBVMCU has been discussed in the dedicated paragraph. PTLPDs are discussed in detail in part 3 of the review; however, the correct knowledge of the clinical context (post-transplant setting) is essential for PTLD diagnosis.

Despite being rare, primary GI cHL should also be kept in mind because of its possible EBV expression. Unlike cHL, EBV-positive DLBCL, NOS shows a complete B-cell phenotype [8].

PBL is a possible differential diagnosis as PBL predominantly occurs at extra-nodal sites, including GIT [8,59,60]. In PBL, the large cells resembling immunoblasts (IBs) or plasmablasts (PBs) generally show a plasmacytic phenotype with expression of CD138, CD38 and MUM1/IRF4, whereas CD20 is virtually not expressed in contrast with EBV-positive DLBCL, NOS. EBER is positive in most PBL with a predominance of type I latency pattern in contrast with type II and type III in EBV-positive DLBCL, NOS. The presence of *IGH/MYC* translocation should suggest PBL, as *MYC* translocation is absent in EBV-positive DLBCL, NOS.

Primary effusion lymphoma (PEL) is a rare, aggressive B-cell neoplasm arising in individuals who are either immunocompromised or elderly [61]. In its classic form, PEL involves body cavities, presenting with effusions.

The extra-cavitary variant, presenting with solid masses, needs to be considered in the differential diagnosis with EBV-positive DLBCL, NOS [62].

Unlike the classic form, which is usually negative for B-cell markers, EC-PEL, often expressing some B-cell markers, may resemble EBV-positive DLBCL, NOS. However, unlike EBV-positive DLBCL, NOS, the presence of Kaposi Sarcoma Herpes Virus/Human Herpes Virus 8 (KSHV/HHV8) represents a diagnostic criterion for PEL. PEL affecting HIV-positive individuals is usually coinfected by HHV8 and EBV, whereas in HIV-negative cases, PEL may lack EBV infection [8].

LYG is a rare EBV-driven clonal LPD, generally occurring in individuals with an underlying immunodeficiency [8,63,64]. It is mostly an extra-nodal disease, affecting predominantly the lungs [8,63,64]. In absence of lung involvement, a diagnosis of LYG should be considered very unlikely. Other common sites, often involved synchronously with the lungs, include the brain, liver, kidneys and skin. GI involvement is uncommon [8,63,64,65]. LYG consists of a variable but often low number of EBV-positive B-cells, with an angiocentric and angiodestructive pattern, within a reactive background in which T lymphocytes predominate. The disease grading is based on the number of EBV-positive cells. Unlike EBV-positive DLBCL, NOS, LYG grade 3 lacks a diffuse pattern of growth, and it usually shows necrosis around vessels and angioinvasion by CD4-positive T lymphocytes [8,63,64,65].

In the present review, we do not discuss diffuse large B-cell lymphoma associated with chronic inflammation (DLBCL-CI) and fibrin-associated diffuse large B-cell lymphoma (FA-DLBCL), both of which are associated with EBV infection, as, to the best of our knowledge, no cases involving the GIT have been reported so far [8,66,67].

### 4.6. Treatment and Oucome

Patients with EBV-positive DLBCL, NOS have a worse prognosis when compared with patients with its EBV-negative counterpart; EBV expression represents, per se, an adverse prognostic factor [45]. Patients with IS present more often with intestinal localization and higher clinical stage [45]. Cases with upregulation of PD-L1 and PD-L2 show worse prognosis than PD-L1/PD-L2 negative cases. Patients receive immune-chemotherapy, usually with the R-CHOP regimen (rituximab, cyclophosphamide, doxorubicin, vincristine, prednisone). However, immunomodulatory therapies targeting the axis PD-1/PD-L1 as well as treatments targeting the JAK-STAT and NF-κ pathways may represent therapeutic options [47,48].

## 5. cHL

### 5.1. General Features and Etiology

cHL is a clonal lymphoid neoplasm characterized by neoplastic elements (mononuclear Hodgkin cells and multinucleated Reed Sternberg cells (RSCs)) of B-cell origin, in the context of a reactive, inflammatory background. Based on the characteristics of the reactive infiltrate and on the morphology of the neoplastic cells, the disease is divided into four subtypes:

Nodular sclerosis cHL (NSCHL), lymphocyte-rich cHL (LRCHL), mixed cellularity cHL (MCCHL) and lymphocyte-depleted cHL (LDCHL) [8].

The association with EBV varies among the subtypes, being MCCHL and LDCHL more often EBV-positive (75% of cases). Both epidemiology and clinical presentation vary across the subtypes.

NSCHL, the most common subtype, is usually a disease of young adults, often presenting as a mediastinal mass [8].

LRCHL occurs in patients older than those with other subtypes and with a male predominance; it is usually a nodal disease, with rare mediastinal involvement and patients are often in low stages (I or II).

MCCHL and LDCHL are found more frequently in developing countries and in HIV-positive individuals. MCCHL shows two peaks of incidence, in young adults and elderly, predominantly males. It is usually a nodal disease and mediastinal involvement is infrequent; B symptoms are common. LDCHL is the rarest subtype, more frequent in males of different ages (from 30 to 71 years). Sites often involved are retroperitoneal lymph nodes, abdominal organs and BM. Patients with LDCHL are more likely to present with B symptoms and advanced stage disease. Prognosis of MCCHL and LDCHL is worse than other subtypes, although patients may benefit from intensive therapeutic strategies [8].

### 5.2. cHL and GIT

Primary extra-nodal cHL represents a rare event (less than 1% of HL cases) [8,68]. Among the extra-nodal sites, the GIT is the most commonly involved. Primary GI cHL represents less than 5% of GI lymphomas [21,69,70,71,72,73,74]. The majority of GI cHL are secondary involvement of the GIT in the context of a systemic disease.

The primary nature of the disease in GIT is established after a complete patient work-up confirming that the disease is limited to the GIT with or without local lymph node involvement. In order to exclude a secondary spread to the GIT from another site, involvement of peripheral lymph nodes and/or BM, liver or spleen should not be present.

Primary GI cHL can present either as tumor-forming lesion involving the whole thickness of the GI wall, or as stricture or ulcerated lesion [21,69,70,71,72,73,74].

Primary cHL of the GIT has been reported in association with conditions of IS and, in particular, in the context of IBD [75,76,77], although observed even in immunocompetent patients [21,78].

The group of Elaine Jaffe reported a series of primary GI cHL arising in the setting of longstanding IBD or chronic inflammation [75]. It has been hypothesized that immunologic defects associated with IBD might predispose IBD patients to develop GI cHL; however, azathioprine-induced IS could play a relevant role in some cases of IBD-associated cHL [75]. The cases of cHL, arising in IBD, resulted to be EBV-positive, similarly to cHL occurring in other conditions of IS, such as HIV infection and after transplantation [8]. In the setting of IS, EBV plays a possible causative role in the pathogenesis of cHL [75]. In retrospect, some of these EBV-associated cHL arising in IBD patients resemble, at least in part, EBVMCU. Therefore, it is possible that at least some of the previously reported GI cHL could be now reconsidered as EBVMCU. cHL and EBVMCU are likely to be different points in the spectrum of an EBV-related LPD [21].

### 5.3. Histology, Immunophenotype and Genetic Profile

**Figure 3:**
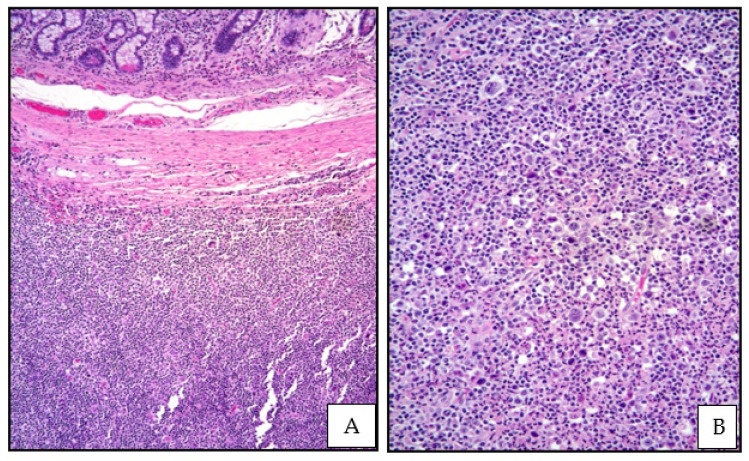
(**A**) cHL: medium power view showing a dense lymphoid infiltrate involving the small bowel wall (Hematoxylin and eosin, magnification 100×, original image from Prof S.A.); (**B**) cHL: high power of the polymorphic lymphoid infiltrate containing large-sized cells with Hodgkin features (Hematoxylin and eosin, magnification 200×; original image from Prof S.A.).

The morphological features of cHL vary across the different subtypes. The neoplastic elements, Hodgkin cells and bi-or multinucleated RSCs, are in variable number and admixed to non-neoplastic inflammatory cells, including small lymphocytes, eosinophils, plasma cells and histiocytes (Figure 3).

Epithelioid histiocytes forming granulomas may be present. Broad bands of collagen are usually present in NSCHL [8].

**Figure 4:**
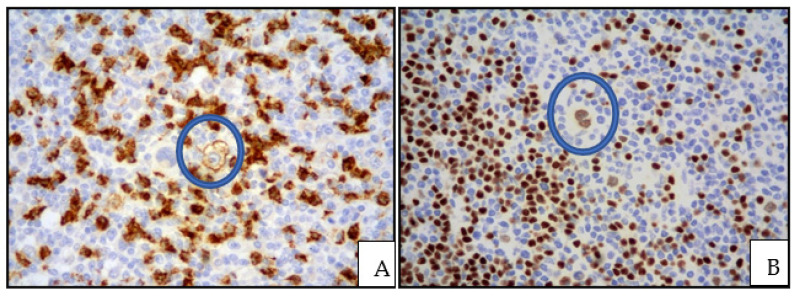
(**A**) cHL: CD15 expression highlighting a Hodgkin cell with membranous positivity (inside the blue circle); in the background, granulocytes are strongly CD15 positive (immunostaining, magnification 400×; original image from Prof S.A.); (**B**) cHL: weak PAX5 positivity of a Hodgkin cell, inside the blue circle; unlike Hodgkin cells, B lymphocytes strongly express PAX5 (immunostaining, magnification 400×; original image from Prof S.A.).

The neoplastic cells express CD30 in nearly all cases, whereas CD15 is expressed in a lesser percentage of cases (Figure 4).

**Figure 5:**
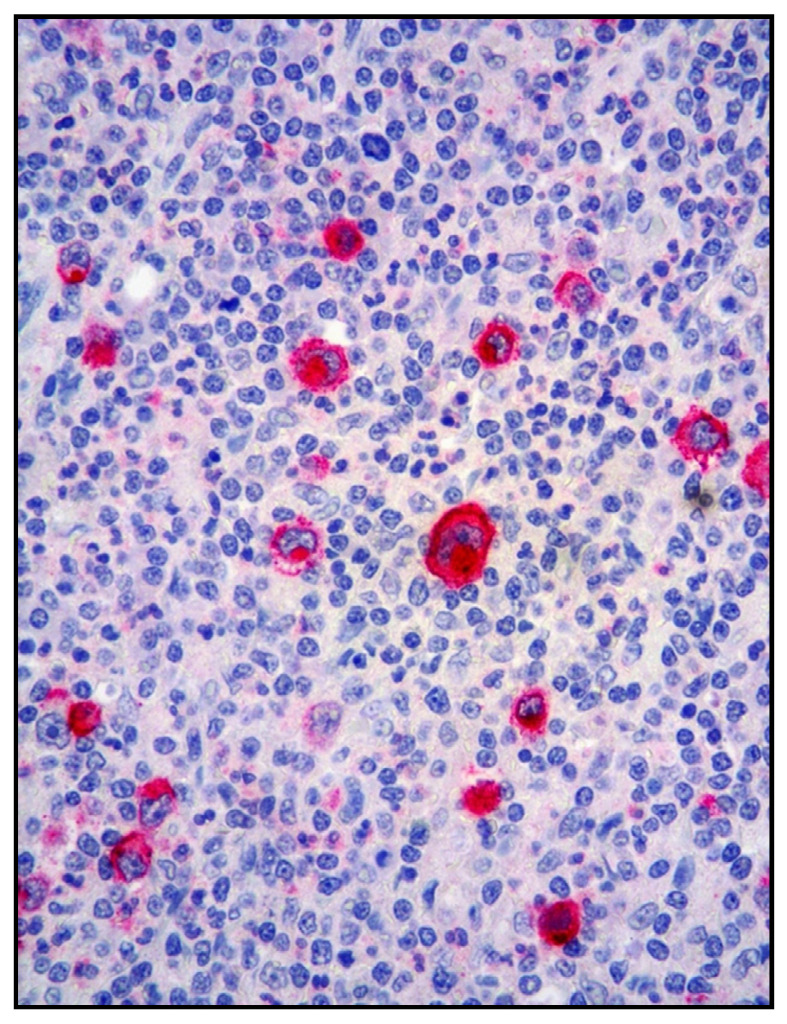
cHL: LMP1 expression in Hodgkin cells (immunostaining, magnification 400×; original image from Prof S.A.).

PAX5 is generally weakly positive, whereas B-cells markers such as CD20 and CD79alpha are either negative or weakly positive only in a subset of neoplastic cells. CD45 is usually negative. OCT2 and BOB1 are often negative; however, either OCT2 or BOB1 may be positive, but generally not both [8]. Aberrant expression of T-cell markers can be observed. The association with EBV is demonstrated by EBER or LMP1 expression (Figure 5).

EBV association is found more frequently in MCCHL (75% of cases) than in NSCHL (10–25%).

TCR gene rearrangement is usually negative, whereas IGH gene rearrangement is frequently positive, especially in cases rich in neoplastic elements [8].

### 5.4. Differential Diagnosis

Due to the rarity of primary GI cHL, a high degree of caution is advisable when considering cHL in the differential diagnosis of a GI lesion. Stringent criteria should be applied before making this diagnosis. The distinction from EBVMCU can be complex, but it is relevant due to the different outcome of these entities and therapeutic implications.

Of note, primary GI cHL is mainly a mass-forming lesion, whereas EBVMCU is typically a superficial and circumscribed ulcer [21]. This differential diagnosis is discussed in more detail in the section on EBVMCU.

Strong expression of B-cell markers such as CD20 and CD79alpha makes unlikely the diagnosis of cHL and should prompt consideration of EBV-positive DLBCL, NOS, in which Hodgkin cells and RSCs may be present [79].

### 5.5. Treatment and Outcome

Prognosis and treatment strategies for cHL are mainly based on stage disease and histology [80].

cHL is curable in about 80% of cases. Patients with early-stage disease are treated with abbreviated courses of chemotherapy, usually with ABVD scheme (doxorubicin, bleomycin, vinblastine and dacarbazine), followed by involved-field radiotherapy.

Patients with advanced stage disease usually receive a longer course of chemotherapy, generally without radiotherapy. The anti-CD30 Brentuximab Vedotin is also currently used. Checkpoint inhibitors such as anti-PD-1 are promising therapeutic options. cHL is very responsive to PD-1 blockade due to genetic alterations in 9p21.1, leading to PD-L1 high expression [81]. Considering the virally linked increased expression of PD-L1, EBV-associated lymphomas are often sensitive to PD-1 blockade. Relapsed or refractory disease is generally treated with high-dose chemotherapy followed by autologous stem cell transplant (auto-SCT).

## 6. Conclusions

EBV-driven LPDs of the GIT represent an expanding spectrum of diseases often arising in conditions of IS, ranging from localized and often self-limiting forms to aggressive lymphomas.

As entities with different biological behavior may share overlapping features, in routine practice, these disorders represent diagnostic and therapeutic challenges.

A strict correlation among clinical data, macroscopic, histological and genetic findings is essential to deliver the correct diagnosis and adequate treatment.

## Data Availability

Individual patient data from the original studies included in the present review is not available and data sharing at this level is not applicable for a systematic review.
